# Pathogenicity Studies of NADC34-like Porcine Reproductive and Respiratory Syndrome Virus LNSY-GY and NADC30-like Porcine Reproductive and Respiratory Syndrome Virus GXGG-8011 in Piglets

**DOI:** 10.3390/v15112247

**Published:** 2023-11-11

**Authors:** Hechao Zhu, Liuqing Wei, Xiangzu Liu, Shudan Liu, Huanchun Chen, Pin Chen, Xiangmin Li, Ping Qian

**Affiliations:** 1 National Key Laboratory of Agricultural Microbiology, Hubei Hongshan Laboratory, Huazhong Agricultural University, Wuhan 430070, China; yxbigsuper@gmail.com (H.Z.); wlq-987215@webmail.hzau.edu.cn (L.W.); liuxiangzu@webmail.hzau.edu.cn (X.L.); vet2439134082@163.com (S.L.); chenhch@mail.hzau.edu.cn (H.C.); lixiangmin@mail.hzau.edu.cn (X.L.); 2 College of Veterinary Medicine, Huazhong Agricultural University, Wuhan 430070, China; chenpin@mail.hzau.edu.cn; 3 The Cooperative Innovation Center for Sustainable Pig Production, Wuhan 430070, China

**Keywords:** PRRSV, NADC30-like, NADC34-like, pathogenicity

## Abstract

The porcine reproductive and respiratory syndrome virus (PRRSV) has caused significant economic losses to the swine industry. The U.S., China, and Peru have reported NADC30-like or NADC34-like PRRSV-infected piglets, which have been identified as the cause of a significant number of abortions in clinics. Although the pathogenicity of NADC30-like PRRSV and NADC34-like PRRSV in piglets exhibits significant variability globally, studies on their pathogenicity in China are limited. In this study, the animal experiments showed that within 8–14 days post-infection, both piglets infected with NADC30-like PRRSV GXGG-8011 and those infected with NADC34-like PRRSV LNSY-GY exhibited significant weight loss compared to the control piglets. Additionally, the viremia of the LNSY-GY persisted for 28 days, while the viremia of piglets infected with the GXGG-8011 lasted for 17 days. Similarly, the duration of viral shedding through the fecal–oral route after the LNSY-GY infection was longer than that observed after the GXGG-8011 infection. Furthermore, post-infection, both the LNSY-GY and GXGG-8011 led to pronounced histopathological lesions in the lungs of piglets, including interstitial pneumonia and notable viral colonization. However, the antibody production in the LNSY-GY-infected group occurred earlier than that in the GXGG-8011-infected group. Our research findings indicate that LNSY-GY is a mildly pathogenic strain in piglets, whereas we speculate that the GXGG-8011 might be a highly pathogenic strain.

## 1. Introduction

The porcine reproductive and respiratory syndrome virus (PRRSV) is a significant economic burden in global pork production [1]. It is estimated that the U.S. pork industry spends at least USD 60 billion annually [2]. This enveloped, positive-sense, single-stranded RNA virus belongs to the family *Arteriviridae*, genus *Betaarterivirus*, according to the International Committee on Taxonomy of Viruses (ICTV) [3]. The PRRSV possesses a complex genome, approximately 15,000 nucleotides (nt) in length, and accommodates more than 10 open reading frames (ORFs), including ORF1a, ORF1b, ORF2a, ORF2b, ORF3-7, and ORF5a [4,5].

Based on the differences in geographic origin and genetic variation, the PRRSV is typically classified into two species: PRRSV type 1 (PRRSV 1, European genotype) represented by the Lelystad virus (LV) strain, and the ATCC VR-2332 strain, representing PRRSV type 2 (PRRSV 2, North American genotype) [6]. Prior to 2017, there were three main subtypes of the PRRSV prevalent in China, which included the classic strains CH-1a and S1; the highly pathogenic PRRSV (HP-PRRSV) strains JXA1, HuN4, and TJ; and the NADC30-like PRRSV strains JL580, CHsx1401, and HNjz15 [7,8]. Considering the ORF5 gene, genotype 2 can be further divided into nine sublineages [9]. The NADC30-like PRRSV belongs to sublineage 1.8, while the NADC34-like PRRSV falls within sublineage 1.5 [10]. NADC30-like PRRSV infections were first reported in pig farms across several Chinese provinces in 2015, followed by NADC34-like PRRSV infections that emerged in 2017, with the genetic origin of both viruses traced back to the U.S. [8,11,12]. Under experimental conditions, both the NADC30-like PRRSV and NADC34-like PRRSV exhibited wide-ranging virulence in U.S. and China’s pig farms [13,14]. Song et al. isolated the NADC34-like PRRSV strain HLJDZD32-1901 from Heilongjiang Province, China, confirming its moderate pathogenicity in piglets [15]. However, Yuan et al. isolated the NADC34-like PRRSV strain JS2021 from Jiangsu Province, China, resulting in piglets infected with this strain exhibiting persistent fever, weight loss, and higher rates of illness and mortality, confirming its high pathogenicity in piglets [16].

Given the substantial differences in pathogenicity between the NADC30-like and NADC34-like PRRSV in piglets, we conducted comparative pathogenicity experiments using the NADC34-like PRRSV LNSY-GY strain and the NADC30-like PRRSV GXGG-8011 strain, both in China. This research aims to provide experimental evidence for the epidemiology and transmission of NADC30-like PRRSV and NADC34-like PRRSV, which can increase awareness of the prevention and control of those viruses.

## 2. Materials and Methods

### 2.1. Viruses and Cells

The NADC34-like PRRSV LNSY-GY strain was isolated from Liaoning Province, and the NADC30-like PRRSV GXGG-8011 strain was isolated from the Guangxi Zhuang Autonomous Region in China. Porcine alveolar macrophages (PAMs) were obtained from 4-week-old specific pathogen-free (SPF) piglets and cultured in an RPMI 1640 medium (Gibco BRL Co., Ltd., Irvine, CA, USA) supplemented with 10% fetal bovine serum (Gibco BRL Co., Ltd., USA) at 37 °C in 5% CO_2_. The viral supernatants were collected from infected PAM cells at the indicated time points and titrated with TCID_50_ and reverse transcription real-time quantitative PCR (qPCR).

### 2.2. Sequencing Alignment and Phylogenic Analysis 

For phylogenetic analysis, the sequences of the ORF5 gene and NSP2 amino acid of PRRSV strains in different lineages/sublineages were aligned using the Clustal W method and muscle method using the Molecular Evolutionary Genetics Analysis Version 7.0 (MEGA 7) software, respectively. The sequence homology of the nucleotide and amino acid was determined using the MegAlign software 7 (Lasergene, DNASTAR, Madison, WI, USA). A phylogenetic tree was constructed from the aligned nucleotide sequences using the neighbor-joining method with a bootstrap value of 1000 replicates. 

### 2.3. Viral Growth Kinetics of PRRSV

A one-step growth curve study was performed in PAM cells to ascertain the growth characteristics of the NADC34-like PRRSV LNSY-GY strain and NADC30-like PRRSV GXGG-8011 strain at a 0.01 multiplicity of infection (MOI). The cell culture supernatant was detected via TCID50 after serial tenfold dilution and detected via PMAs at different time points like 12, 24, 36, 48, 60, 72, 84, 96, 108, and 120 h post-infection (hpi).

### 2.4. Animals and Experimental Design

Fifteen 8-week-old female piglets (Duroc–Yorkshire–Landrace crossbred commercial pigs), free from the porcine reproductive and respiratory syndrome virus (PRRSV), the pseudorabies virus (PRV), the classical swine fever virus (CSFV), and the porcine circovirus 2/3 (PCV2/3), were randomly divided into three groups (the pigs were randomly assigned to the three groups using the “Random Drawing” method, ensuring that the assignment process was not influenced by any characteristics of the pigs). Each pig’s identification No. was written on individual cards. These cards were then placed in a box, thoroughly shuffled, and drawn one by one to assign the pigs to three groups, each containing five piglets (specific pathogen detection primers are shown in Table 1). Piglets in the first group were infected with the NADC34-like PRRSV LNSY-GY at 4 × 10^5^ TCID_50_/pig via intranasal (2 mL/nasal) and intramuscular (2 mL) routes simultaneously. In the second group, pigs were simultaneously infected with the NADC30-like PRRSV GXGG-8011 via intranasal (2 mL/nasal) and intramuscular (2 mL) routes, with an infectious dose of 4 × 10^5^ TCID_50_/pig. The third group of piglets received an equivalent volume of DMEM via the same routes as the placebo. Following viral infection, the piglets underwent daily monitoring for their rectal temperature and clinical signs, and the body weights of piglets were measured at 0, 7, 14, and 28 days post-infection (dpi). Clinical signs that included lethargy, anorexia, skin discoloration, sneezing, coughing, labored and abdominal breathing, and respiratory rate were graded using a scoring system as previously reported [17]. They were euthanized in a humane manner either when they exhibited moribund conditions or at the end of the study, which was terminated at 28 dpi. All animal experiments received approval from the Institutional Animal Care and Use Committee and strictly adhered to conventional animal welfare regulations and standards.

### 2.5. Detection of PRRSV via qPCR

Blood was collected at 0, 3, 7, 10, 14, 17, 21, 24, and 28 dpi for viremia detection via qPCR. Nasal and rectal swabs were collected and analyzed using the same method as above. During necropsy, samples were collected from the heart, liver, spleen, lung, kidney, brain, cerebellum, brainstem, submandibular lymph nodes, mesenteric lymph nodes, inguinal lymph nodes, duodenum, jejunum, ileum, cecum, colon, rectum, tonsils, ovaries, and uterus for virus detection using qPCR. The total RNA was extracted from serum samples and tissue samples using an RNeasy mini-kit (NECVB, Harbin, China) according to the manufacturer’s instructions. The qPCR was performed as described previously. An absolute quantitative system was prepared with FastKing (TIANGEN, Harbin, China) one-step reagents and amplified using LightCycler^®^ 480 Instrument II (Roche, Basel, Swiss Confederation). Briefly, this method uses specific primers and fluorogenic probes based on the 3′UTR gene of the PRRSV (forward primer: 5′-ATRATGRGCTGGCATTC-3′; reverse primer: 5′-ACACGGTCGCCCTAATTG-3′; and TaqMan probe: 5′-HEX-TGTGGTGAATGGCACTGATTGACA-BHQ2-3′). The length of the amplified fragment was 112 bp. For samples, approximately 1 g of tissue was weighed and homogenized with 2 mL of DMEM. The tissue samples were homogenized at 25 Hz for 20 min (Qiagen Tissue Lyser II, Hilden, Germany) and diluted with sterile PBS; the collected supernatant was followed by centrifugation at 8000× *g* for 10 min at 4 °C to remove residual tissue debris. A total of 200 μL of the supernatant of each tissue and serum sample was used for RNA extraction. Then, 6 μL of RNA was used for qPCR analysis. qPCR was used to quantify the copy numbers of the PRRSV genomic cDNA. A recombinant plasmid containing the 3′UTR gene of the PRRSV was used for the construction of a standard curve. The plasmid was serially diluted 10-fold, with concentrations ranging from 10^2^ to 10^10^ copies/mL. 

### 2.6. Serological Test 

Blood samples of piglets were collected at 0, 3, 7, 10, 14, 17, 21, 24, and 28 dpi for the detection of a PRRSV-specific antibody. Anti-PRRSV antibodies were detected using an ELISA kit (JNT PRRS X3, Beijing, China) according to the manufacturer’s instructions. The PRRSV-specific antibody titer was reported as sample-to-positive (S/P) ratios. The serum samples with an S/P ratio of 0.4 or higher were considered positive. 

A neutralization assay was performed as previously described with minor modifications. Briefly, an inactivated serum (56 °C, 30 min) was serially diluted in a 2-fold ratio, and a 30 μL (from 2^−1^ to 2^−8^) serum was mixed with the 30 μL 200 TCID_50_ NADC30-like PRRSV or NADC34-like PRRSV virus at 37 °C for 2 h and repeated four times. Then, separately added to the monolayer of PAM cells in a 96-well plate. After 5 days, the titers of the PRRSV-specific neutralizing antibody were calculated and expressed as log2 of the reciprocal of the highest dilution of when PAM cell infection inhibited a cytopathic effect (CPE).

### 2.7. Histopathological to Lungs 

Macroscopic or histopathological scoring was performed as previously reported [18]. The sum of all frequencies was an estimation of the percentage of the affected lung on a scale from 0 to 100 [19]. Briefly, each lung lobe was designated a numeric value representing the approximate volume percentage of the entire lung it occupied. Specifically, ten points, divided into five for the dorsal aspect and five for the ventral aspect, were allocated to the right anterior lobe, the right middle lobe, the anterior section of the left anterior lobe, and the caudal portion of the left anterior lobe. Additionally, the accessory lobe received 5 points, while the right and left caudal lobes were assigned 27.5 points each, consisting of 15 for dorsal and 12.5 for ventral. This cumulative scoring system added up to 100 points in total. Gross lung lesion scores were estimated, and a score was given to reflect the amount of pneumonia in each lobe. The total points for all the lobes were an estimate of the percentage of the entire lung with grossly visible pneumonia. Tissue samples were fixed in formalin for subsequent histopathological analysis using hematoxylin and eosin staining. To be more specific, in each lung lobe, we scored interstitial pneumonia based on the following scale: 0, indicating an absence of microscopic lesions; 1, representing mild interstitial pneumonia; 2, indicating moderate multifocal interstitial pneumonia; 3, suggesting moderate diffuse interstitial pneumonia; and 4, denoting severe interstitial pneumonia. The total score, with a maximum possible value of 4 points, was interstitial pneumonia. The lung samples were submitted to additional immunohistochemistry (IHC) with a specific monoclonal antibody against the PRRSV N protein (Zoonogen M100022, Beijing, China) to detect the distribution of the PRRSV antigen in the lung samples according to those previously reported [20]. The remaining tissues were stored at −80 °C.

### 2.8. Statistical Analysis

All the statistical analyses were performed using GraphPad Prism software.6 Comparisons among different groups were evaluated using one-way ANOVA. Data were expressed as the mean ± the standard errors (SE). In all cases, *p* < 0.05 was considered a statistically significant difference.

## 3. Results

### 3.1. Viral Kinetics and Nsp2 and ORF5 Sequence Analysis of NADC30/34-like PRRSV

**Figure 1:**
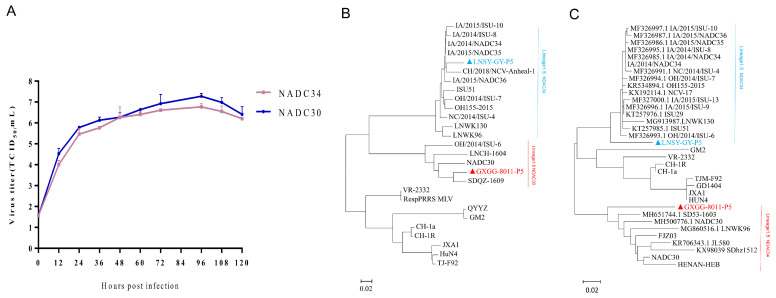
(**A**) The viral kinetics of the two PRRSV strains used in this study. Sequence alignment and phylogenetic analysis of the PRRSV. Phylogenetic analysis PRRSV based on the Nsp2 (**B**) and the ORF5 (**C**) gene. GXGG-8011 labeled with 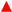 belongs to sublineage 1.8 (NADC30-like), while LNSY-GY labeled with 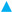 belongs to sublineage 1.5 (NADC34-like).

At a multiplicity of infection (MOI) of 0.01, the virions of the NADC30-like strain GXGG-8011 in the culture supernatant reached a peak of 1.0 × 10^7.27^ TCID_50_/mL at 96 hpi. Subsequently, there was a slight reduction in the viral titer to 1.0 × 10^6.41^ TCID_50_/mL by 120 hpi. In contrast, the virions of the NADC34-like strain LNSY-GY in the culture supernatant reached a peak of 1.0 × 10^6.77^ TCID_50_/mL at 96 hpi, with a slight reduction in the titer to 1.0 × 10^6.20^ TCID_50_/mL by 120 hpi (Figure 1). Therefore, our study indicates that infecting PAM cells at 0.01 MOI for 96 h represents the optimal choice for achieving higher viral yields, with the NADC30-like GXGG-8011 strain exhibiting higher proliferative titers compared to the NADC34-like strain LNSY-GY.

**Figure 2:**
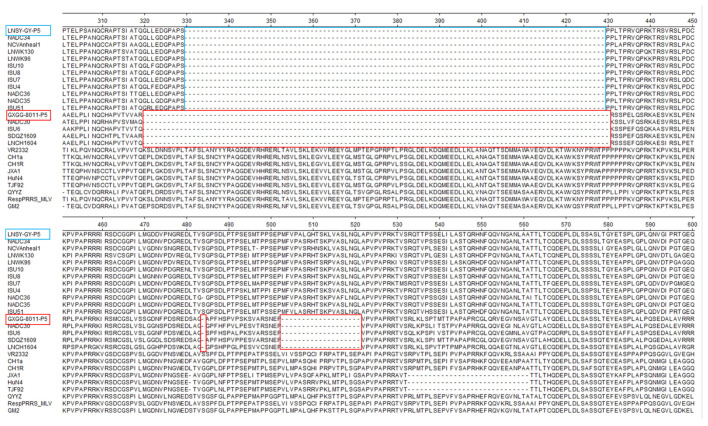
Sequence alignment of NSP2 proteins. The GXGG-8011 exhibits an identical deletion pattern, as observed in the NADC30-like strains, which encompass 131 amino acids, ranging from aa320–430, aa483, and aa503 to aa521 (111 + 1 + 19), labeled with a red box. The LNSY-GY and NADC34-like PRRSV shared the same 100-aa deletion, labeled with a blue box, corresponding to positions 330–429 in the VR2332 NSP2 protein.

The Nsp2 and ORF5 regions are the most variable regions in the genome of the PRRSV, which is widely recognized for conducting systematic evolutionary and genetic variation analyses of the PRRSV internationally. We generated phylogenetic trees based on Nsp2 nucleotide sequences and ORF5 nucleotide sequences, respectively. As shown in Figure 1, compared to the LNSY-GY clustered into lineage 1.5, which represented NADC34-like, the GXGG-8011 clustered into lineage 1.8, representing NADC30-like. As shown in Figure 2, compared to the Nsp2 of VR2332, the Nsp2 protein of the GXGG-8011 exhibits an identical deletion pattern, as observed in the NADC30-like strains, which encompass 131 amino acids, ranging from aa320–430, aa483, and aa503 to aa521 (111 + 1 + 19) (Figure 2); while the Nsp2 protein of the LNSY-GY had a deletion pattern that was identical to those of the NADC34-like strains, all of them had a deletion of 100 amino acids at aa330–429 (Figure 2). The accession numbers for the ORF5 and Nsp2 genes were uploaded to the NCBI public database (GXGG-8011-P5 ORF5: OR758910, LNSY-GY-P5 ORF5: OR758911; GXGG-8011-P5 NSP2: OR758912; and LNSY-GY-P5 NSP2: OR758913).

### 3.2. Clinical Presentations of Piglets after PRRSV Infection

**Figure 3:**
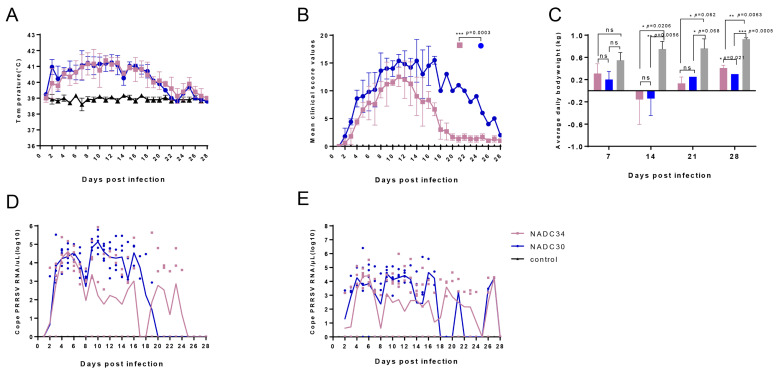
Rectal temperatures, average daily body weights, and oral–fecal detoxification in the experimental piglets. (**A**) Body temperature changes in piglets infected with the PRRSV. A rectal temperature ≥ 39.5 °C was defined as fever. The mean ± SD (error bars) of temperatures is shown. (**B**) Mean clinical score during the PRRSV infection. (**C**) Body weight changes during the PRRSV infection. The body weight gain of piglets was calculated at 7, 14, 21, and 28 dpi. The mean ± SD (error bars) of body weight gain is shown. *, *p* < 0.05, **, *p* < 0.01, ***, *p* < 0.001. ns, no significant difference. (**D**,**E**) Virus shedding patterns after the PRRSV infection. PRRSV RNA copies in oral–nasal (**D**) and fecal of (**E**) piglets were detected using real-time qPCR.

Following the challenge, the control group showed no apparent clinical signs throughout the entire experimental period. In contrast, piglets infected with the PRRSV exhibited clinical symptoms such as dehydration, respiratory distress, and shivering. After viral infection, whether with NADC30-like GXGG-8011 or NADC34-like LNSY-GY strains, the piglets experienced elevated temperatures exceeding 39.5 °C from 2 dpi to 20 dpi, returning to normal at 22 dpi (Figure 3A). The control group of piglets maintained consistent normal body temperatures. Clinical signs that included lethargy, anorexia, skin discoloration, sneezing, coughing, labored and abdominal breathing, and respiratory rate were graded using a scoring system. The results revealed that piglets infected with NADC30-like showed significantly higher average clinical scores than the NADC34-like group in 28 days (*p* = 0.0003, *p* < 0.001, Figure 3B).

We measured the body weights of piglets in each group daily to assess the impact of the PRRSV infection on body weight across four different time periods: 0–7 dpi, 8–14 dpi, 15–21 dpi, and 22–28 dpi. As shown in Figure 3C, piglets infected with the GXGG-8011 and the LNSY-GY exhibited a notable decline in weight from day 8 dpi to 14 dpi. From 8 dpi to 28 dpi, there was a significant difference in weight changes compared to the control group (*p* < 0.01). Following the conclusion of the experiment, piglets infected with the LNSY-GY exhibited a daily weight gain of 0.409 kg/day, which was significantly lower than the control group’s daily weight gains, exceeding 0.9 kg/day (*p* = 0.0063, *p* < 0.01). Similarly, piglets infected with the GXGG-8011 had a daily weight gain of 0.3 kg/day, which was also significantly lower than the control group (*p* = 0.0005, *p* < 0.001). These findings suggest that the LNSY-GY-infected group had a higher weight gain compared to the GXGG-8011-infected group (*p* = 0.021, *p* < 0.05) (Figure 3B).

To evaluate the shedding of the PRRSV in piglets after infection, we collected daily samples to analyze the viral shedding patterns in nasal and fecal samples. In the GXGG-8011-infected group, nasal shedding ceased at 20 dpi, while fecal shedding continued until 21 dpi. In contrast, the LNSY-GY-infected group stopped nasal shedding at 25 dpi, with fecal shedding persisting until 28 dpi. It is worth noting that the quantity of virus shed through the nasal route after PRRSV infection exceeded that through the fecal route, and the GXGG-8011-infected group exhibited a shorter shedding duration compared to the LNSY-GY-infected group (Figure 3D,E). In summary, these data indicate that clinical harm inflicted on piglets after infection with the LNSY-GY is milder compared to the clinical harm caused by the GXGG-8011 infection.

### 3.3. Viremia Examination and Serological Test Analysis 

**Figure 4:**
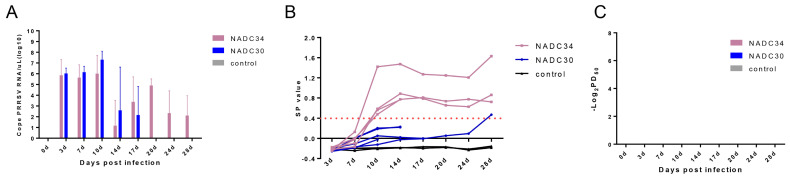
Viremia examination and serological test analysis. (**A**) Dynamics of viremia were detected using qPCR. (**B**) Pig serum was analyzed for PRRSV-specific antibodies. The threshold for seroconversion was set at a sample-to-positive (S/P) ratio of 0.4. (**C**) Pig serum was analyzed for PRRSV neutralization antibodies. The bars represent the average S/P of one group of piglets. The mean ± SD (error bars) of the specific antibodies is shown.

Viremia could be detected in all infected piglets from 3 dpi. At the same time, the NADC30-like GXGG-8011-infected group had a shorter duration of viremia compared to the NADC34-like LNSY-GY-infected group, the GXGG-8011-infected group exhibited a higher level of viral shedding, and no viremia was detected in the serum samples from piglets in the control group throughout the experimental period (Figure 4A). Piglets in the NADC34-like LNSY-GY-infected group were seroconverted (S/P > 0.4) at 10 dpi, in contrast to the GXGG-8011-infected group, where the immune response appeared at 28 dpi, and antibodies in the control group remained negative throughout the experiment (Figure 4B). Additionally, we also assessed the presence of neutralizing antibodies against the PRRSV in the serum of the infected group. Nevertheless, as of 28 dpi, neither of these viral infections elicited the production of neutralizing antibodies (Figure 4C). These data indicate that LNSY-GY generates antibodies earlier compared to GXGG-8011, and the latter results in a milder viral viremia.

### 3.4. Pathogenicity and Viral Loads in Tissues

**Figure 5:**
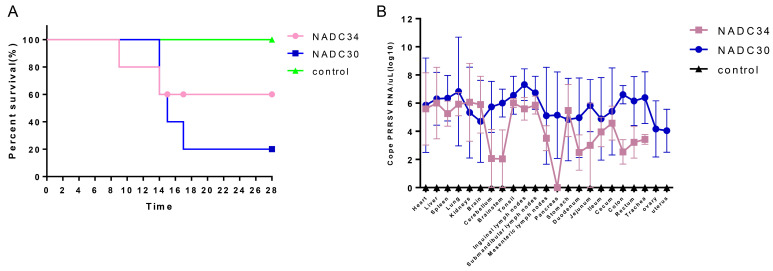
The pathogenicity of the PRRSV in piglets. Viremia (**A**) and viral loads in tissues (**B**) were measured using qPCR. Each bar represents the average for one group of piglets ± SD.

The experiment concluded with a survival rate of 20% (1/5) for piglets in the NADC30-like GXGG-8011 infected group, while piglets in the NADC34-like LNSY-GY infected group displayed a 60% survival rate (3/5), clearly indicating that NADC30-like GXGG-8011 exhibited greater pathogenicity in piglets compared to NADC34-like LNSY-GY (Figure 5A). All surviving piglets no longer exhibited any clinical signs of the PRRSV and were then humanely euthanized at 28 dpi. During necropsy, infected piglets exhibited severe lung consolidation and necrosis, along with hemorrhage and necrosis in the tonsils and lymph nodes, with no significant changes noted in these tissues in the control group. The subsequent stage involved conducting measurements of tissue viral loads to evaluate the extent of damage caused by viral infections. The findings revealed that the GXGG-8011-infected group exhibited higher viral content in various organs compared to the LNSY-GY-infected group. Notably, viral RNA remained undetectable in samples collected from the piglets in the control group (consult Figure 5B). It is essential to recognize that the widespread distribution of the virus throughout the body after infection with GXGG-8011 and LNSY-GY could potentially pose a continued threat to pig production.

### 3.5. Histopathological and Immunohistochemistry Examinations

**Figure 6:**
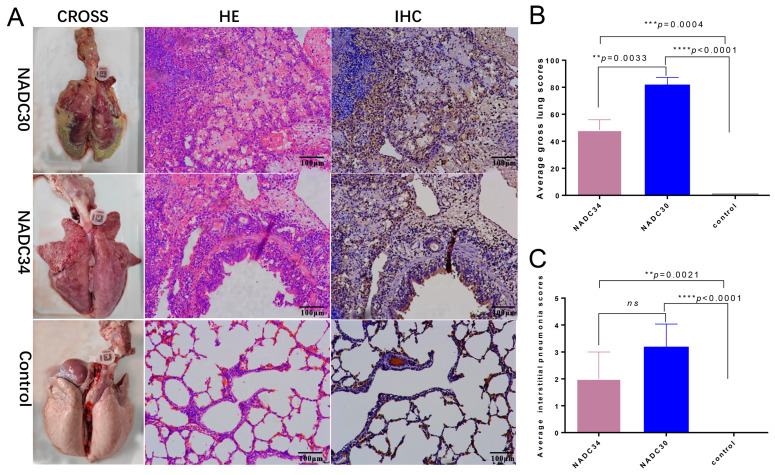
Gross, histological lesions and immunohistochemistry of lungs from the three groups of piglets. (**A**) Consolidation and ecchymosis in the lungs were compared with those of controls (**left panels**). Infiltration of inflammatory cells around bronchiole, alveolar septa, and alveolar spaces (**middle panels**); extensive serous and interstitial pneumonia in the lungs were compared with that of the control (**middle panels**). Positive brown-red macrophages in the lungs were compared with those of the control (**right panels**). (**B**) The difference in average lung gross scores between the lungs of the control and the PRRSV-infected animals was presented. (**C**) The difference in average interstitial pneumonia scores between the lungs of the control and the PRRSV-infected animals was presented, **, *p* < 0.01, ***, *p* < 0.001, ****, *p* < 0.0001. ns, no significant difference.

During the necropsy, lung consolidation was observed in both NADC30-like GXGG-8011-infected piglets and NADC34-like LNSY-GY-infected piglets but not in animals from the control group (Figure 6A left panels). Histopathological examination was next performed to evaluate the tissue damage caused by the PRRSV infection. Microscopic examination revealed that piglets in both infected groups displayed interstitial pneumonia; no microscopic pathological lesions were detected in the lungs of piglets from the control group (Figure 6A middle panels). Furthermore, PRRSV antigens in the lungs of the infected piglets were examined using immunohistochemistry. A monoclonal antibody specific to the nucleocapsid protein of the PRRSV was used in immunohistochemistry (IHC) staining to reveal the presence of a viral antigen in the tissues. As shown in Figure 6A (right panels), the lungs of all piglets in the PRRSV-infected groups were full of PRRSV-positive signals; no PRRSV-positive signals were seen in the lungs of control piglets. The parenchyma of the NADC30-like infected lungs exhibited increased firmness and weight compared to the NADC34-like infected lungs. The average lung lesion score in the NADC30-like group was significantly higher (*p* < 0.001) than that in the NADC34-like-infected group and the control group (as shown in Figure 6B). In addition, we conducted an assessment of interstitial pneumonia resulting from infections with these two strains. The results demonstrated that NADC30-like infection induced more severe interstitial pneumonia compared to NADC34-like infection, as depicted in Figure 6C.

## 4. Discussion

During the early stages of pregnancy, the PRRSV is acknowledged to induce embryonic mortality [21]. However, the clinical manifestations of a PRRSV infection typically become apparent later in gestation, leading to outcomes such as abortion, fetal death, and the birth of weak offspring [22]. Furthermore, in Chinese swine herds, gastrointestinal problems are frequently observed in conjunction with PRRSV infections, often leading to reduced food consumption and weight loss [23]. An analysis of clinical data reveals a significant increase in sow abortions during NADC30-like and NADC34-like PRRSV outbreaks, indicating that these strains tend to exhibit relatively high pathogenicity in gilts and sows, frequently resulting in abortion [24]. 

In 2013, the NADC30-like PRRSV emerged in China, resulting in significant economic losses [25,26]. Moreover, the NADC30-like PRRSV rapidly emerged as the prevailing strain in China, exceeding the infection rate of HP-PRRSVs in many Chinese pig farms post-2016 [27]. The PRRSV 1-7-4 (NADC34-like) strain made its initial appearance in the U. S. back in 2014 and was designated as IA/2014/NADC34 [28]. From 2015 to 2017, multiple PRRSV 1-7-4 (NADC34-like) strains were also identified in Peru [29]. Between 2017 and 2021, NADC34-like strains spread to 10 different Chinese provinces, surging from 3% in 2017 to 11.5% in 2020 and a remarkable 28.6% in 2021, with the most significant prevalence noted in Heilongjiang Province [30,31,32], and it is evident that NADC34-like strains are more prevalent in northern China than in the southern regions [13,14,31]. More recently, the complete genome of a NADC34-like strain was reported in South Korea, and the strain was the result of a recombination between NADC34-like (major parent) and NADC30-like (minor parent) [33]. Likewise, in 2017, two NADC34-like strains (named LNWK96 and LNWK130) were isolated in Liaoning, China, and genomic sequencing revealed these to be chimeric viruses [34]. 

The virulence of NADC30-like and NADC34-like PRRSV exhibits significant variations in pathogenicity [35]. Recent pathogenicity studies on NADC30-like viruses have indicated that they generally cause milder symptoms and lower mortality rates compared to HP-PRRSV isolates [26,35,36]. Similarly, Ma et al. reported that NADC30-like PRRSV induces severe intestinal infections and demonstrates tropism in piglets [37]. In 2021, the PRRSV strain RFLP 1-4-4 Lineage 1c Variant (NADC34-like), which caused high mortality in piglets and finishing piglets, was reported in the U.S., affecting most pig farms in the Midwestern U.S. [38]. Yuan et al. observed that pigs infected with JS2021NADC34 PRRSV experienced prolonged fever, reduced body weight, high morbidity, and mortality, underscoring the high pathogenicity of the NADC34 PRRSV in pigs [16]. Liu et al. noted the emergence of PRRSV/CN/FJGD01/2021, a recombinant virus arising from NADC30-like, NADC34-like, and JXA1-like isolates, of which isolated from sows during a period of high abortion rates (20%) in China in 2021, has intermediate virulence for piglets [39]. 

To further investigate the pathogenicity of Chinese NADC30-like and NADC34-like PRRSV strains, we conducted pathogenicity tests on two strains: NADC30-like GXGG-8011, isolated from the Guangxi Zhuang Autonomous Region, China, in 2021, and NADC34-like LNSY-GY, isolated from Liaoning Province, China, in 2021. Eight-week-old piglets infected with both viruses exhibited typical clinical symptoms, including high fever, respiratory issues, and weight reduction. Piglets infected with the GXGG-8011 displayed more severe clinical manifestations compared to pigs infected with the LNSY-GY. Infection with the GXGG-8011 resulted in a longer duration of viremia and shedding through the fecal–oral route compared to infection with the LNSY-GY. Furthermore, the intermittent shedding of the PRRSV after infection can be attributed to a variety of factors, including i. Fluctuations in Immune Responses: Immune responses within pigs can fluctuate, leading to instability in viral clearance [40]; ii. Complex Infection Dynamics: PRRSV infection dynamics are intricate, involving multiple rounds of viral replication and dissemination within the pig’s body, which can result in the virus reappearing at different time points [10]; iii. Immune Suppression: PRRSV has the ability to suppress the pig’s immune system, making it challenging for the immune system to effectively clear the virus, contributing to recurrent infections [41]; iv. Infection and Shedding Cycles: The cycles of PRRSV infection can lead to viral shedding at different time points [22,42].

Additionally, the NADC34-like LNSY-GY induced antibody production by 10 dpi, whereas antibodies were detected only by 28 dpi in piglets infected with the GXGG-8011. This suggests that the LNSY-GY triggers the host’s antibody response earlier than the GXGG-8011. Neutralizing antibodies (nAbs) are essential components of the immune defense against viral infections [43]. However, as of 28 dpi, neither virus induced the production of neutralizing antibodies, which is consistent with other studies showing that the PRRSV does not typically stimulate the production of neutralizing antibodies within the first 28 dpi [42,44,45]. However, Qiu et al. found that PRRSV-specific heterologous neutralizing antibodies (shnAbs) could be detected as early as 42 dpi (2/6 pigs) and reached their peak at 63 dpi (4/6 pigs), after which they declined. This indicates the need for additional research incorporating serological monitoring after infection [45]. Furthermore, infection with the GXGG-8011 led to a higher mortality rate of only 80% (4/5), whereas the LNSY-GY provided a lower mortality rate of 60% (3/5). Clearly, the former resulted in a higher piglet mortality rate after infection. Histopathologically, the GXGG-8011 infection resulted in more severe lung lesions than the LNSY-GY, which aligns with recent reports indicating that NADC30-like or NADC34-like strains induce severe histopathological manifestations [15]. All these findings suggest that while the NADC34-like LNSY-GY PRRSV is virulent to pigs, it exhibits lower pathogenicity compared to the NADC30-like GXGG-8011 PRRSV strain.

Nonetheless, it is crucial to acknowledge that secondary infections can significantly exacerbate losses in clinical scenarios. This holds particularly true because the clinical severity consistently exceeds that observed in controlled animal experiments in real-world clinical practice. PRRSV infections can heighten the host’s vulnerability to additional pathogens such as *Streptococcus suis*, *Haemophilus parasuis* (*H. parasuis*), *Salmonella choleraesuis*, and *Mycoplasma hyopneumoniae*. Secondary infections in the field stand as one of the primary contributors to more severe PRRSV symptoms. In addition, the importance of host genetics in the manifestation of disease due to the PRRSV (porcine reproductive and respiratory syndrome virus) infection is substantial and multifaceted (Different pig breeds or genetic lines exhibit varying levels of susceptibility to the PRRSV. Some genetic backgrounds are more resistant to the virus, while others are more susceptible. Pigs with specific genetic backgrounds may manifest more severe symptoms, higher mortality rates, or prolonged viremia compared to others. Genetic factors influence the severity of respiratory distress, reproductive issues, and the overall impact of the disease. The duration and intensity of viral shedding can be influenced by host genetics. Certain pigs may shed the virus for extended periods, which heightens the potential for viral transmission to other pigs.) [46,47]. Researchers have conducted extensive research on the pig genome and viral infections, yielding novel insights for PRRSV prevention, disease control, and the development of disease-resistant breeding strategies [48,49]. Hence, the significance of prevention and control measures for the NADC30-like and NADC34-like PRRSV cannot be overstated. We strongly advocate for continuous surveillance and further research on the NADC30-like and NADC34-like PRRSV, as these endeavors are pivotal for effective management.

## 5. Conclusions

All these findings suggest that while the NADC34-like LNSY-GY PRRSV is virulent to pigs, it exhibits lower pathogenicity compared to the NADC30-like GXGG-8011 PRRSV strain.

## Figures and Tables

**Table 1:** The primers used to detect specific pathogens in our study.

Virus	Prime	Sequence (5′-3′)	Size (bp)
PRV	gE-F	GCTGTACGTGCTCGTGAT	101
	gE-R	TCAGCTCCTTGATGACCGTGA	
	gE-Probe	HEX-CACAACGGCCACGTCGCCACCTG-BHQ1	
	CSFV-F	TACAGGACAGTCGTCAGTTCGA	
CSFV	CSFV-R	CCGCTAGGGTTAAGGTGTGTCT	92
	CSFV-Probe	FAM-CCCACCTCGAGATGCTATGTGGACGA-TAMRA	
PCV2	PCV2-F	CACGGATATTGTAGTCCTGGT	500
	PCV2-R	CGCACCTTCGGATATACTGTC	
PCV3	PCV3-F	TTACTTAGAGAACGGACTTGTAACG	645
	PCV3-R	AAATGAGACACAGAGCTATATCAG	

## Data Availability

Data are contained within the article.
